# Knowledge and perceptions about malaria in communities in four districts of the Central African Republic

**DOI:** 10.1186/s13104-015-1124-x

**Published:** 2015-04-19

**Authors:** Gustave Bobossi Serengbe, Jean-Methode Moyen, Rosine Fioboy, Edith Narcisse Beyam, Cyriaque Kango, Colette Bangue, Alexandre Manirakiza

**Affiliations:** University of Bangui, PO Box 1450, Bangui, Central African Republic; Complexe Pédiatrique de Bangui, Ministry of Public Health, Population and AIDS Control, PO Box 883, Bangui, Central African Republic; Malaria Programme Division, Ministry of Public Health, Population and AIDS Control, PO Box 883, Bangui, Central African Republic; United Nations Population Fund, Bangui, PO Box 873, Bangui, Central African Republic; Institut Pasteur of Bangui, PO Box 923, Bangui, Central African Republic

**Keywords:** Malaria, Knowledge, Community, Central African Republic

## Abstract

**Background:**

Implementation of malaria control strategies may face major social and cultural challenges. Hence, understanding local knowledge about malaria helps in designing sustainable community-based malaria control programmes. We designed a pilot survey in communities in the Central African Republic to evaluate recognition of malaria symptoms, perceptions of the causes of malaria and knowledge of key preventive measures.

**Methods:**

This cross-sectional study was conducted in four districts. Households were selected by multi-stage cluster random sampling, with villages (in Lobaye, Ouham and Ouaka) and boroughs (in Bangui City) as first-stage units and households as second-stage units. A total of 2920 householders were interviewed.

**Results:**

Most of the respondents attributed malaria to mosquito bites (65.5%), but less than 50% were familiar with the classical symptoms of malaria. Hygiene and sanitation were the most frequently mentioned methods for preventing malaria (81.1%). Despite the relatively high rate of ownership of insecticide-treated nets (72.1%), community perception of these nets as a preventive measure against mosquito bites was very low (6.5%).

**Conclusions:**

The correct perceptions that mosquitoes cause malaria transmission and of environmental management for prevention are encouraging; however, awareness about the usefulness of insecticide treated-nets for malaria prevention must be raised. This study provided the national malaria control programme with baseline data for planning appropriate health education in communities.

## Background

Malaria is a major public health problem, especially for vulnerable groups such as children under 5 years and pregnant women in sub-Saharan Africa, where 90% of malaria deaths occur [1,2]. In 1997, leaders at the annual meeting of the Organization of African Unity called for action to control malaria, and a global partnership to “roll back” malaria was initiated [3]. In 1998, the World Health Organization, the World Bank, the United Nations Development Programme and the United Nations Children’s Fund formed the Roll Back Malaria (RBM) partnership. One of the aims of RBM is to provide all families with health information to ensure effective prevention and effective first-line treatment of malaria for young children [4].

The Abuja Declaration in 2000 called on all African Member States to commit themselves to reduce the malaria burden by half by the year 2010. The targets of the Declaration were reduction of the malaria burden by at least 60% and of overall mortality by 50%, at least 60% of people with malaria having access to prompt treatment with antimalarial drugs and at least 60% of those at risk, particularly children under 5 and pregnant women, benefiting from community protective measures such as insecticide-treated nets [5]. The targets also include increasing the percentage of children with fever who receive adequate treatment and increasing the prevalence of use of bed nets by high-risk populations [6].

Implementation of malaria control strategies requires adequate synergy between service delivery and community response [7,8]. The strategies may face major social and cultural challenges that negatively influence the choice, acceptance and use of malaria control interventions [9-11]. A number of studies have been conducted about knowledge and perceptions relating to malaria in Africa, which found that misconceptions concerning malaria are still common [12-15]. Understanding local knowledge about malaria can help in designing sustainable community-based malaria control programmes that will lead to behavior change and adoption of new ideas and technology [11].

The Central African Republic (CAR) is a vast, sparsely populated country, covering 623 000 km^2^ and with a population in 2006 of 4 299 502 inhabitants. The national malaria control programme implemented the first phase of RBM activities during the period before 2005–2009, which consisted of achieving significant health care coverage and sensitization of the population about preventive aspects. So far, no study on knowledge and perception about malaria has been conducted in the country. Hence, the present study was undertaken to assess recognition of malaria symptoms, perceptions about the causes of malaria and knowledge of preventive measures in the community.

## Method

### Study area

**Figure 1:**
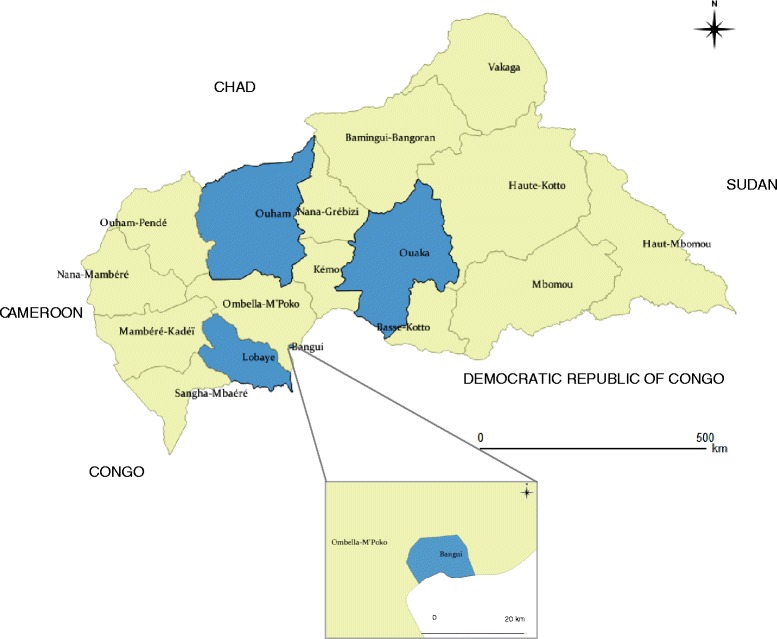
Study locations.

A survey was conducted during October 2009 in households in four districts, Lobaye, Ouham, Ouaka and Bangui City (Figure 1), selected by the National Division of Malaria Control as part of its efforts to assess achievement of the targets established for implementation of RBM activities.

The climate of the CAR is subequatorial, with a rainy period between April and November and temperatures varying from 19°C to 32°C. Malaria transmission is perennial; all areas of the country are exposed to endemic malaria, with a peak during the rainy season, although no data are available on the intensity of malaria transmission. Malaria accounts for more than 40% of morbidity in the country (CAR Ministry of Health, unpublished data). The activities of the national malaria programme consist of implementing RBM strategies to reduce morbidity and mortality related to malaria in the general population by reaching the coverage rate recommended in the Abuja Declaration [5].

### Training

A team of 10 investigators received 12 days (8 h/day) of training in participant sampling techniques, conducting interviews and filling in questionnaires. A pilot survey was conducted to assess the validity of the questionnaire and the skills of the investigators in collecting information in an unselected region (Ombela M’Poko) during June 2009.

### Sample size and sampling procedure

Households were selected by multi-stage cluster random sampling, with villages (in Lobaye, Ouham and Ouaka) and boroughs (in Bangui City) as first-stage units and households as second-stage units. In each region, we selected 30 villages or boroughs after probability proportional sampling based on the number of inhabitants. A list of villages (in Lobaye, Ouham and Ouaka) and boroughs (in Bangui City) and the population size of each area were obtained from the national census [16]. The formula *n* = (*d* (*z*_α/2_)^2^*p* (1–*p*)/ *i*^2^ was used to calculate the sample sizes of householders required. Calculations were based on 50% (*p*) malaria prevention and treatment indicators [17], *z*_α/2_ = 1.96, a 4% margin of error (*i*), a design effect of 1.5 (*d*) and a 20% non-response rate. Hence, a sample of 1035 householders was estimated for each region. Random selection of households was achieved with the following formula: 1035*(*H*_*v*_*/H*_*t*_), where *H*_*v*_ is the number of households in each village or borough and *H*_*t*_ is the total number of households in the selected village or borough.

### Data collection and analysis

The investigators were distributed as follows: three in Ouham, three in Ouaka, two in Bangui and two in Lobaye, who used cars or motorbikes to reach each survey point. The house of the village or borough chief was considered as the starting-point, and random direction by flipping a coin was used to select subsequent households for interview. A household was defined as the entity in which people live together and have a meal from a common cooking facility, and a householder was defined as the person who is perceived by members of household as the key decision-maker in the family [18]. Data on causes, symptoms and prevention of malaria were collected on a standard questionnaire during an interview constructed in French and translated into Sango (the local language). The questionnaire was in a closed-ended format, with some questions allowing open-ended answers.

The data collected were entered onto an Excel spreadsheet and analysed with SPSS software version 11. Descriptive statistics were used to measure frequencies and percentages of variables.

### Ethical approval and informed consent

The study received ethical approval from the institutional review boards of the CAR Ministry of Health. Informed consent was obtained from all study participants. The heads of the communities were involved in population sensitization to participate in the survey.

## Results

A total of 2920 householders were interviewed (70.5% of the expected sample size). Table 1 shows the distribution of the study population according to region. Householders reported disparate perceptions of the causes and symptoms of malaria. Most attributed the disease to cold weather during the rainy season (67.5%) and to mosquito bites (65.6%); others associated malaria with intestinal worms (32.0%), fatigue (3.7%) and sorcerers or evil spirits (3.4%). Most respondents in Bangui (79.7%) and in Ouaka (79.0%) attributed malaria to cold weather, while 73.3% of respondents believed that mosquito bites cause malaria transmission in Ouham, 72.0% in Ouaka, 63.3% in Lobaye and 56.2% in Bangui.

**Table 1:** **Knowledge of respondents about the causes and symptoms of malaria according to survey site, Central Africa Republic, 2009**

		**Lobaye (n = 589)**	**Ouham (n = 731)**	**Ouaka (n = 685)**	**Bangui (n = 915)**	**All (N = 2920)**
Cause	Mosquito bite	63.3	73.3	72.0	56.2	65.5
	Cold weather	59.6	48.0	79.0	79.7	67.5
	Intestinal worms	35.0	43.2	20.1	29.9	32.0
	Fatigue	1.4	4.7	3.5	4.6	3.7
	Sorcerer, evil spirits	4.1	1.2	3.2	4.9	3.4
Signs and symptoms	Abdominal pain	53.5	68.3	72.0	64.9	65.1
	Yellowish urine	46.5	38.2	28.5	47.0	40.3
	Fever	42.6	17.1	44.1	50.8	39.1
	Convulsions	40.1	36.3	61.5	42.4	44.9
	Muscle pain	20.4	45.4	27.2	37.3	33.5
	Diarrhoea	24.4	13.8	29.6	26.0	23.5
	Chills	6.3	6.7	6.4	12.3	8.3
	Pallor	5.4	1.4	9.5	5.7	5.4
	Vomiting	1.4	3.8	2.8	5.1	3.5
	Headache	2.7	3.8	3.1	3.8	3.4

Abdominal pains, convulsions, yellowish urine and fever were the most frequently mentioned symptoms, reported by 65.1%, 44.9%, 40.3% and 39.1% of respondents, respectively.

Hygiene and sanitation were the most frequently mentioned methods for preventing malaria (81.1%), while only 6.5% of participants believed that insecticide-treated nets provided protection from mosquito bites, although 72.1% of households reported that they had been given a net (Table 2).

**Table 2:** **Ownership of insecticide-treated nets and knowledge about prevention of malaria according to survey site, Central Africa Republic, 2009**

**Variable**	**Lobaye (n = 589)**	**Ouham (n = 731)**	**Ouaka (n = 685)**	**Bangui (n = 915)**	**All (N = 2920)**
Own an insecticide-treated net	71.5	73.3	69.3	73.6	72.1
Knowledge of preventive measures					
Hygiene and sanitation	67.9	83.3	87.2	84.3	81.1
No insecticide-treated net	10.0	19.2	15.4	14.7	13.1
Chloroquine	3.6	7.7	17.9	17.6	12.0
Insecticide-treated net	1.8	3.8	12.8	8.8	6.5

## Discussion

More than half the householders mentioned mosquito bites as the mode of malaria transmission. In malaria-endemic areas, people are aware of the role of mosquito bites in the transmission of malaria; our result is lower than those reported in most studies from other sub-Saharan countries [11,13,19,20], but higher than that reported by Paulander *et al*. [21] in Ethiopia and by Eyobo *et al*. [22] in Sudan. Our finding agrees with that reported by Singh *et al*. [23] in Nigeria.

Despite the relatively high level of awareness about the mode of malaria transmission, some people still believed that exposure to cold weather is a direct cause of malaria (malaria is known in Sango as *kobela ti de* or “cold disease”). The idea might have stemmed from the concurrence of cold, cloudy weather and the presence of mosquito breeding sites. The perception of respondents that intestinal worms and fatigue are causes of malaria is surprising, although fatigue could be related to symptoms of malaria, and, given the shared endemicity of malaria and soil-transmitted helminths, these diseases often co-exist in the same populations. Helminths may therefore be associated with a continued and possibly increased incidence of malaria infection [24-26]. This evidence corroborates the high rate of reporting of abdominal pain as a symptom of malaria. The socio-anthropological belief of some respondents that malaria is associated with sorcery may be due to the belief in sub-Saharan Africa that convulsions (a symptom of severe malaria) are due to evil spirits [27].

The relatively low rate (<50%) of respondents who were familiar with the classical symptoms of malaria is a cause for concern, because it may delay treatment. Other studies reported that respondents had good knowledge about malaria signs and symptoms [18,23].

Substantial numbers of householders (81.1%) were aware of the importance of hygiene and sanitation for malaria prevention. This finding corroborates those of other studies [19,21]. This is not surprising, because the message “maintaining environmental sanitation” is an important component of the malaria control programme [28-31], and this key sensitization aspect is frequently used in malaria prevention and control interventions in the CAR.

Despite the relatively high rate of ownership of an insecticide-treated net (72.1%), the community perception of these nets as a preventive measure against mosquito bites was very low (6.5%). This result is lower than those reported in other studies: 63.9% in the United Republic of Tanzania [32], 48.7% in Cameroon [19], 46.2% in Ethiopia [21] and 34.0% in Sudan [22]. Adongo *et al*. [11] mentioned awareness of the role of nets in malaria nuisance reduction but not in malaria prevention.

The main limitation of this study is the lack of data on socio-demographic status (age, sex and educational level), as it would be useful to test their influence on correct knowledge about malaria [21,33,34]. In the CAR, it has been argued that each region present its own challenges for implementation of the health programme because of cultural and socio-economic diversity, which is masked by national averages [35]. Hence, comparison of the findings from different study sites would be uninformative, as each locality has social and cultural specificities. Another limitation is that the expected sample size was not reached. The physical inaccessibility of some villages and boroughs due to scarce land transport was a serious constraint in this study.

## Conclusion

This study provided the national malaria control programme with baseline data for planning appropriate health education in communities. The findings showed that perceptions of mosquito bites as the cause of malaria transmission and of environmental management for prevention are satisfactory; however, awareness about the use of insecticide-treated nets as a preventive measure against malaria was poor. Hence, emphasis on sensitizing communities about the use of insecticide treated-nets for malaria prevention is suggested. Core population coverage indicators for RBM are needed.
